# NFAT Signaling in Osteoblasts Regulates the Hematopoietic Niche in the Bone Microenvironment

**DOI:** 10.1155/2013/107321

**Published:** 2013-09-01

**Authors:** Cheryl L. Sesler, Majd Zayzafoon

**Affiliations:** Department of Pathology, University of Alabama at Birmingham, Birmingham, AL 35294, USA

## Abstract

Osteoblasts support hematopoietic cell development, including B lymphopoiesis. We have previously shown that the nuclear factor of activated T cells (NFAT) negatively regulates osteoblast differentiation and bone formation. Interestingly, in smooth muscle, NFAT has been shown to regulate the expression of vascular cellular adhesion molecule-1 (VCAM-1), a mediator of cell adhesion and signaling during leukocyte development. To examine whether NFAT signaling in osteoblasts regulates hematopoietic development *in vivo*, we generated a mouse model expressing dominant-negative NFAT driven by the 2.3 kb fragment of the collagen-*α*I promoter to disrupt NFAT activity in osteoblasts (dnNFAT^OB^). Bone histomorphometry showed that dnNFAT^OB^ mice have significant increases in bone volume (44%) and mineral apposition rate (131%) and decreased trabecular thickness (18%). In the bone microenvironment, dnNFAT^OB^ mice displayed a significant increase (87%) in Lineage^−^cKit^+^Sca-1^+^ (LSK) cells and significant decreases in B220^+^CD19^−^IgM^−^ pre-pro-B cells (41%) and B220^+^CD19^+^IgM^+^ immature B cells (40%). Concurrent with these findings, LSK cell differentiation into B220^+^ cells was inhibited when cocultured on differentiated primary osteoblasts harvested from dnNFAT^OB^ mice. Gene expression and protein levels of VCAM-1 in osteoblasts decreased in dnNFAT^OB^ mice compared to controls. These data suggest that osteoblast-specific NFAT activity mediates early B lymphopoiesis, possibly by regulating VCAM-1 expression on osteoblasts.

## 1. Introduction

The family of nuclear factor of activated T-cell (NFAT) transcription factors is composed of five proteins (NFATc1–c4, NFAT-5) and is known for its role in T-cell development and differentiation [1, 2]. NFAT proteins have also been implicated in the differentiation of other biological systems, including the central nervous system, lungs, heart, integument, skeletal muscle, intestines, and bone [2–4]. In resting cells, NFAT proteins are highly phosphorylated and reside in the cytoplasm. Upon increases in intracellular calcium and the activation of calcineurin (Cn), NFAT is dephosphorylated and translocates to the nucleus, where it acts as a transcriptional regulator of NFAT-dependent genes [2]. 

Previous studies have shown that Cn/NFAT signaling positively regulates osteoblast differentiation and bone formation, but these reports were conducted using animal models with global overexpression or knockout of calcineurin A-alpha and NFATc1 [5–7]. This model does not sufficiently represent the effects of Cn/NFAT signaling in osteoblasts because it is not an osteoblast-specific model. In contrast, and in support of our findings, Zanotti et al. demonstrate that NFAT overexpression in osteoblasts of ROSA mice inhibits the expression of osteoblast gene markers and function [3, 8, 9] confirming that NFAT expression in osteoblasts negatively regulates bone formation and density. In addition, we have previously shown that the pharmacological inhibition and conditional disruption of calcineurin b1 (Cnb1) in osteoblasts increases osteoblast differentiation and bone formation both *in vitro* and *in vivo* [3, 10], suggesting that Cn/NFAT can be a target for therapeutic drugs to treat osteopenic and osteoporotic patients [10, 11].

Osteoblasts are bone-forming cells originating from mesenchymal progenitors and are located along the bone's endosteal surface [12, 13]. In the adult bone marrow microenvironment, hematopoietic stem/progenitor cells (HSPCs) associate closely with osteoblasts or osteoprogenitor cells [14, 15]. Previous studies have shown that HSPCs can activate bone formation [16], while others have shown that an increase in osteoblast number increases the number of self-renewing HSPCs [17]. In contrast, it has been shown that reduced osteoblast numbers in biglycan knockout mice do not have a negative effect on HSPCs [18]. HSPC expansion and self-renewal have been shown to be significantly higher for HSPCs cultured with osteoblasts versus other stromal cell types, signifying the importance of osteoblasts in this niche [19].

Crosstalk between osteoblasts and HSPCs occurs through critical signaling pathways, including the osteoblast secretion of CXCL12 and IL-7 chemokines and the binding of very late antigen-4 (VLA-4) molecules on HSPCs to vascular cellular adhesion molecule-1 (VCAM-1) receptors on osteoblasts [14, 16, 20]. By blocking the signaling pathway with antibodies directed against VLA-4 or VCAM-1, B cell production is decreased [20, 21]. VCAM-1 expression in osteoblasts is known to be transcriptionally regulated by members of the Rel family, such as nuclear factor kappa B (NF-*κ*B) [22]. Monomers of NFAT, a relative to the Rel/NF-*κ*B family of transcription factors, can also bind in specific areas of NF-*κ*B binding domains [2, 22]. Indeed, NFAT has been shown to transcriptionally regulate VCAM-1 expression in endothelial and smooth muscle cells [23, 24]. In addition, human umbilical vein endothelial cells (HUVECs) transfected with constitutively-active NFAT have shown an 18-fold increase in VCAM-1 gene expression, while inhibiting NFAT activation with siRNA decreased VCAM-1 gene expression levels [23]. 

Here, we examine whether NFAT signaling in osteoblasts regulates hematopoiesis in the bone microenvironment. We generated a mouse model expressing a dominant-negative NFAT driven by a 2.3 kb fragment of the collagen-*α*I promoter to disrupt NFAT activation specifically in osteoblasts (dnNFAT^OB^) by inhibiting Ca^2+^-stimulated nuclear translocation of NFAT transcription factors [25]. We demonstrate that the inhibition of NFAT activity in osteoblasts increases the number of HSPCs and decreases the production of B-lineage cells in dnNFAT^OB^ mice when compared to control mice. VCAM-1 gene expression and protein levels were also reduced when NFAT activation was inhibited. These results suggest that NFAT expression in osteoblasts regulates hematopoiesis in the bone marrow microenvironment, possibly by transcriptional regulation of VCAM-1. 

## 2. Methods

### 2.1. ColI-dnNFAT Transgene Generation

A transgene was generated containing a dominant-negative form of NFAT (dnNFAT) driven by a 2.3 kb fragment of the collagen *α*I promoter (ColI). Flag-tagged dnNFAT was excised from pcDNA3 plasmid (provided by Dr. Chi-Wing Chow, Albert Einstein College of Medicine) [25] by double digestion with *HindIII/NotI*. The 0.5 kb dnNFAT fragment was ligated to a blunt-ended *XbaI* site in the pJ251 plasmid containing the ColI promoter [26], placing the promoter sequence upstream of dnNFAT.

### 2.2. Generation of dnNFAT^OB^ Mice

Mice (C57BL/6 background) expressing ColI-dnNFAT (dnNFAT^OB^) were generated by the Transgenic Animal Core in the Center for Metabolic Bone Disease at the University of Alabama at Birmingham. For mouse genotyping, DNA was extracted from tail biopsies, and a 0.55 kb fragment of the ColI-dnNFAT transgene was amplified by PCR according to manufacturer's recommendations (Sigma, St. Louis, MO, USA). Primer sequences for ColI-dnNFAT were ColI-forward, 5′-TGGACTCCTTTCCCTTCCTT-3′, and dnNFAT-reverse, 5′-GAGGTCGGGGAATACCGATAG-3′. Animals lacking ColI-dnNFAT were used as controls. All animal studies were approved by the Institutional Animal Care and Use Committee of the University of Alabama at Birmingham.

### 2.3. Histology and Histomorphometry

Tibiae and femora were harvested from 12-week-old male and female mice. Tibiae were fixed in 10% (v/v) buffered formalin, decalcified in EDTA, embedded in paraffin, and sectioned. Femora were also fixed, embedded in methyl methacrylate, sectioned, and stained with Goldner's trichrome and von Kossa. A region of interest, an area at least 0.5 mm below the growth plate (excluding the primary spongiosa and trabecular-connected cortical bone), was selected and remained constant for all animals. Standard bone histomorphometry was performed using BioQuant image analysis software (R&M Biometrics, Nashville, TN, USA) in the Histomorphometry and Molecular Analysis Core in the Center for Metabolic Bone Disease at the University of Alabama at Birmingham [3, 10, 27].

### 2.4. Immunohistochemistry

Tibiae were deparaffinized and rehydrated, followed by antigen retrieval with heat treatment in 10 mM sodium citrate buffer, pH 6. Endogenous peroxidase activity was quenched using 3% hydrogen peroxide. Samples were blocked 1 h in 5% goat serum (Fc receptor blocker) (Vector Laboratories, Burlingame, CA, USA). Anti-VCAM-1 and anti-NFATc1 (Santa Cruz Biotechnology, Santa Cruz, CA, USA) were diluted in 5% goat serum (1 : 50) and applied to sections overnight at 4°C. Biotin-conjugated secondary antibodies (2 *μ*g/mL) were added, followed by avidin-biotin enzyme reagents. Specimens were incubated in DAB peroxidase substrate 30 s. Tissues were counterstained in Gill's hematoxylin for 5 s, dehydrated, and mounted. Negative controls were processed alongside the examined tissue. Photos were taken at 200x and 400x magnifications using a Nikon DS-Fi1 digital camera.

### 2.5. Alkaline Phosphatase and Von Kossa Staining

Primary osteoblasts were harvested from the calvariae of 1-day-old control and dnNFAT^OB^ mice and differentiated for 7 (alkaline phosphatase) or 21 (von Kossa) days. Cells were fixed in 2% (v/v) paraformaldehyde for 10 minutes and then incubated at 37°C with alkaline phosphatase substrate solution [3]. Mineralization was assessed with von Kossa staining of the cultures (3 minutes UV in 3% w/v AgNO_3_) [3].

### 2.6. Flow Cytometry Analysis

Bone marrow was flushed from tibiae and femora of 12-week-old mice. Red blood cells were lysed with ACK solution (0.15 M NH_4_Cl, 10 mM KHCO_3_, and 0.1 mM Na_2_EDTA). Bone marrow cells (BMCs) were incubated on ice for 1 hour with FITC labeled lineage antibodies directed against integrins *α*M, Gr-1, Ter119, NK1.1, B220, and CD3; PE labeled antibody against Sca-1; and biotinylated labeled antibody against c-Kit for detection of HSPCs. For the detection of B-lineage cells, BMCs were incubated with antibodies directed against B220 (FITC), CD19 (PE), and IgM (Biotin). Biotin-conjugated cells were then incubated for 30 min on ice with a secondary fluorescent-labeled streptavidin (SAV-APC). All antibodies were purchased from eBiosciences (San Diego, CA, USA). Cells were analyzed on a BD LSR II Analytical Flow Cytometer in the Flow Cytometry Core in the Center for AIDS Research at the University of Alabama at Birmingham. Analysis was performed with FlowJo 7.6 software (Tree Star, Ashland, OR, USA).

### 2.7. Cell Culture and Differentiation

MC3T3 E1 preosteoblastic cells were purchased from the American Type Culture Collection (ATCC, Manassas, VA, USA). Cells were transduced with a constitutively active NFATc1 retrovirus [2]. Primary calvarial osteoblasts were isolated from 1-day-old control or dnNFAT^OB^ mice. Calvariae were digested with three sequential collagenase (type II, Worthington, Lakewood, NJ, USA) digestions. Cells were maintained in Eagle's Minimum Essential Medium, *α* modification, containing 10% fetal bovine serum (Atlanta Biologicals, Lawrenceville, GA, USA), 100 units/mL penicillin G, and 100 *μ*g/mL streptomycin (Invitrogen, Grand Island, NY, USA). Osteoblastic induction was performed by supplementing medium with 10 mM *β*-glycerophosphate and 250 *μ*M ascorbic acid-2-phosphate [10].

### 2.8. Protein Extraction

For whole cell lysates, cells were lysed in 1% Nonidet P-40 lysis buffer. Samples were incubated on a rotator at 4°C for 30 min, centrifuged at 12,000 rpm for 10 min at 4°C, and the supernatant protein concentration was measured. For nuclear protein extracts, cells were washed with chilled PBS and centrifuged at 800 ×g for 5 min at 4°C. Nuclei were then isolated by detergent lysis of the cells with a Nonidet P40 lysis buffer containing 10 mM Tris, 10 mM NaCl, 3 mM MgCl_2_, 0.5% Nonidet P40, and 0.56 M sucrose. Nuclei were then treated with a hypotonic solution containing 10 mM HEPES, 1.5 mM MgCl_2_, and 10 mM KCl followed by a 30 min incubation at 4°C in an extraction buffer containing 20 mM HEPES, 20% glycerol, 600 mM KCl, 1.5 mM MgCl_2_, and 0.2 mM EDTA. Nuclei were finally centrifuged at 12,000 ×g for 30 min at 4°C, and the supernatant protein was collected. All solutions were supplemented with protease (2 *μ*L/mL) and phosphatase (10 *μ*L/mL) inhibitors (Sigma) [10]. Supernatant protein concentrations were measured using the Bio-Rad DC protein assay.

### 2.9. Western Blot Analysis

Protein extracts (25 *μ*g/lane) were separated by SDS-PAGE. After electrophoresis, proteins were transferred to a polyvinylidene difluoride membrane, Immobilon-P (Millipore, Milford, MA, USA), using a Bio-Rad wet transfer system. Membranes were then blocked with TBS-Blotto B (Santa Cruz) for 1 h at room temperature and subsequently incubated overnight with antibodies directed against NFATc1, VCAM-1, lamin C, and *β*-actin (Santa Cruz). Signals were detected using a horseradish peroxidase-conjugated secondary antibody and an enhanced chemiluminescence detection kit (ECL; Amersham Biosciences, Pittsburgh, PA, USA) [10].

### 2.10. RNA Extraction and RT-PCR

Total RNA was extracted using the TRIzol method (Invitrogen). 1 *μ*g of RNA was reverse-transcribed using M-MLV reverse transcriptase, and the equivalent of 12.5 ng was used for Syber Green real-time quantitative RT-PCR. The expression of *β*-actin was used for normalization of gene expression values. The primer sequences used in this study include: VCAM-1, forward 5′-GTCGCGGTCTTGGGAGCCTC-3′ and reverse 5′-TGGACCCCTCCGTCCTCACC-3′; CXCL12, forward 5′-GCTCTGCATCAGTGACGGTA-3′ and reverse 5′-CTTCAGCCGTGCAACAATCT-3′; IL-7, forward 5′-GGCACACAAACACTGGTGAACT-3′ and reverse 5′-TGCATCATTCTTTTTCTGTTCCTT-3′ [28]; and *β*-actin as previously published [10].

### 2.11. Osteoblast/Hematopoietic Stem Cell Cocultures

Primary osteoblasts were harvested from calvariae of 1-day-old mice and maintained in minimum essential Eagle's medium, *α*-modification (*α*-MEM) (Sigma), containing 10% fetal bovine serum (Atlanta Biologicals), 100 units/mL penicillin G, and 100 *μ*g/mL streptomycin (Invitrogen) at 37°C with 5% CO_2_. Cultures were treated with 250 ng/mL amphotericin B (Thermo Scientific, Waltham, MA, USA) treatment 24 h after harvest. Once confluent, osteoblastic induction was performed by supplementing medium with 10 mM *β*-glycerophosphate, 50 *μ*g/mL ascorbic acid, and 10^−7^ M dexamethasone [3]. After differentiating for 14 days, Lin^−^Sca-1^+^cKit^+^ HSPCs were sorted from the bone marrow of tibiae and femora of 12-week-old control mice and seeded onto differentiated primary osteoblasts. Following two additional weeks of differentiation (*α*-MEM, supplemented with 10% FBS, 10 mM *β*-glycerophosphate, and 50 *μ*g/mL ascorbic acid), hematopoietic cells were trypsinized, and B220^+^ cells were analyzed by flow cytometry.

### 2.12. Statistical Analyses

All statistical analyses were performed using the Microsoft Excel data analysis program for two-sample *t*-test analysis assuming unequal variances. Statistical analysis of bone histomorphometry was performed with the nonparametric Mann-Whitney test without assuming normal distribution or unequal variances. Experiments were repeated at least three times unless otherwise noted. Values represent the mean ± standard error or ±standard deviation, as indicated.

## 3. Results

**Figure 1:**
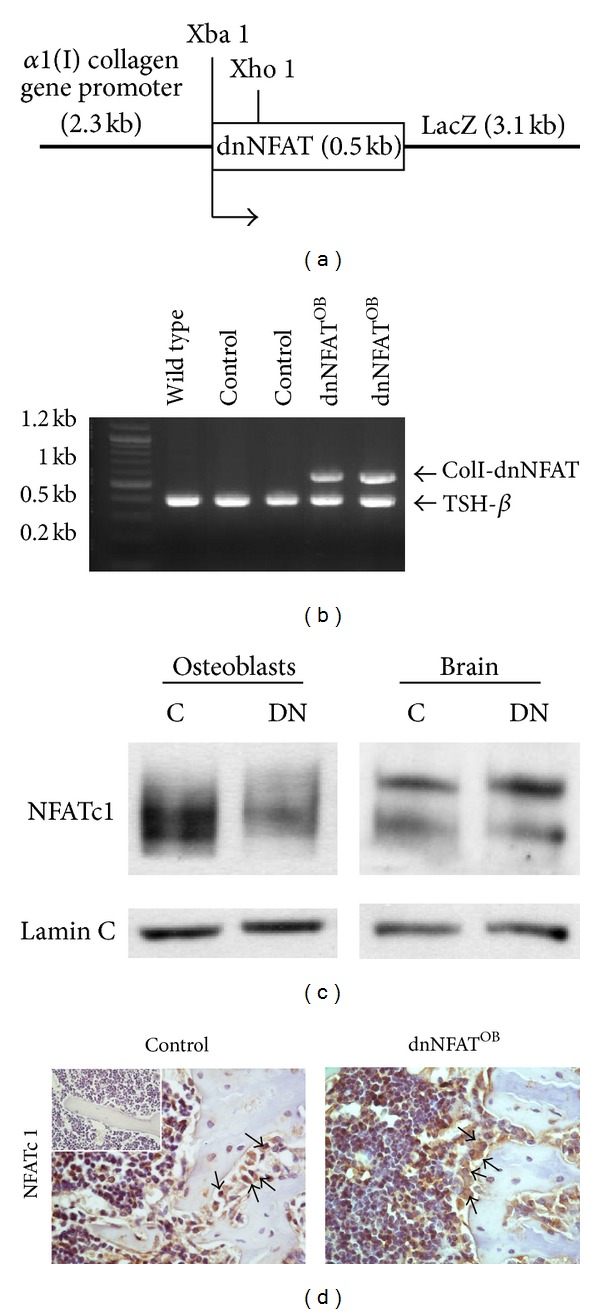
Generation of dnNFAT^OB^ mice. (a) Representation of ColI-dnNFAT transgene construct. (b) DNA was extracted from tail biopsies and amplified by PCR for a 0.55 kb fragment of the ColI-dnNFAT transgene. Mice without ColI-dnNFAT were used as controls. The amplification of thyroid stimulating hormone-beta (TSH-*β*) was used as a loading control. (c) Primary osteoblasts were harvested from calvariae of 1-day-old mice and differentiated 7–14 days. Brain tissue was removed from 12-week-old mice and homogenized. Nuclear proteins were used for immunoblotting with antibodies against NFATc1 and lamin C. Immunoblots are representative of three independent experiments (*n* = 3). (C = control; DN = dnNFAT^OB^.**) **(d) Femora were harvested from 12-week-old control (*n* = 3) and dnNFAT^OB^ (*n* = 3) mice and examined by immunohistochemistry with anti-NFATc1 (*brown*), counterstained with hematoxylin (*blue*). Negative control staining was performed by using normal rabbit IgG instead of primary antibody (*left panel inset*). Magnification, 400x.

To determine whether NFAT signaling in osteoblasts regulates hematopoiesis in the bone marrow microenvironment, we generated ColI-dnNFAT transgenic mice (dnNFAT^OB^) expressing dominant-negative NFAT driven by a 2.3 kb fragment of the collagen *α*1 promoter (Figure 1(a)). Transgenic dnNFAT^OB^ mice were mated with wild-type C57BL/6 mice, and the genotype of offspring was determined by extracting DNA from tail biopsies and performing PCR for detection of the 0.55 kb ColI-dnNFAT transgene (Figure 1(b)). Littermates without ColI-dnNFAT were used as control mice.

To confirm that the NFAT activation is specifically disrupted in osteoblasts, nuclear proteins were extracted from brain tissue and cultured primary osteoblasts. We used NFATc1 as a representative marker for NFAT activation because we and others have previously shown that NFATc1, specifically, is critical for osteoblast differentiation [10]. The dominant-negative NFAT that we used in this study has previously been shown to inhibit the transactivation of NFAT 1–4 isoforms [25]. Our data confirm that NFATc1 translocation is inhibited in osteoblasts and is not altered in the brain tissue from dnNFAT^OB^ mice (Figure 1(c)). Tibiae from 12-week-old mice were sectioned, and immunohistochemistry was performed using antibodies directed against NFATc1. As shown in Figure 1(d), nuclear translocation of NFATc1 decreased, while cytoplasmic protein levels of NFATc1 increased in dnNFAT^OB^ transgenic mice when compared to control mice. 

**Figure 2:**
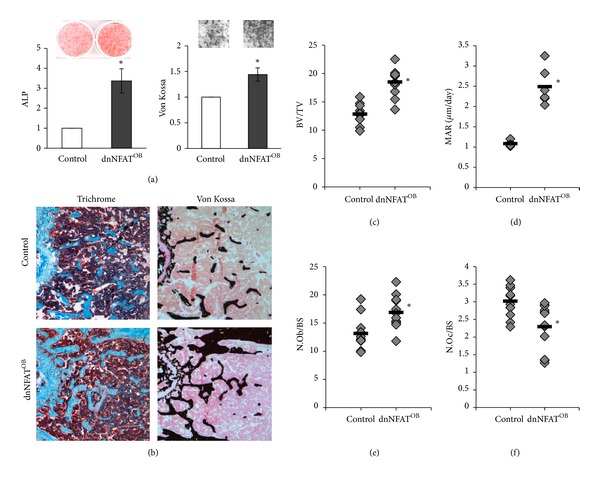
Inhibition of NFAT activation in osteoblasts increases osteoblast differentiation and bone formation. (a) Primary osteoblasts were harvested from the calvariae of control and dnNFAT^OB^ mice and cultured for 7 (alkaline phosphatase, ALP) or 21 (von Kossa) days in the presence of *β*-glycerophosphate and ascorbic acid-2-phosphate to induce osteoblast differentiation. Cells were stained for ALP activity (*red*) or for mineralization by von Kossa (*black*). Images are representative of three independent experiments, each repeated in duplicate. (b) Femora from 12-week-old control and dnNFAT^OB^ mice were stained with Goldner's Trichrome (*blue*) and von Kossa (*black*) staining to show mineralized bone. Magnification, 200x. (c–f) Histomorphometrical indices were measured from 12-week-old control (*n* = 6) and dnNFAT^OB^ (*n* = 6) mice and show (c) bone volume/tissue volume (BV/TV), (d) mineral apposition rate (MAR) (*μ*m/day), (e) osteoblast number/bone surface (N.Ob/BS), and (f) osteoclast number/bone surface (N.Oc/BS). Values represent individual mouse measurements with the mean indicated by the black bar; *P ≤ 0.01 when compared to control mice.

To examine the effects of NFAT inhibition on osteoblast differentiation, primary osteoblasts were isolated from 1-day-old mice and differentiated for 7–21 days. Compared to control, alkaline phosphatase activity (red staining) and mineralization (black) were increased (~3-fold and ~45%, resp.) in dnNFAT^OB^ primary osteoblasts (Figure 2(a)). To examine bone volume in dnNFAT^OB^ mice, femora were isolated from 12-week-old mice and examined by Goldner's trichrome and von Kossa staining for visualization of mineralized bone. Trichrome staining shows that dnNFAT^OB^ mice had an increase in mineralized bone (blue) (Figure 2(b), *left panel*). Von Kossa staining also showed increased bone mineralization (black) in dnNFAT^OB^ mice (Figure 2(b), *right panel*). Bone parameters were quantified using standard histomorphometry and showed significant increases of trabecular bone volume to tissue volume (BV/TV) (44% increase, Figure 2(c)), mineral apposition rate (131% increase, Figure 2(d)), and osteoblast number (28% increase, Figure 2(e)) in dnNFAT^OB^ mice when compared to control mice. Significant decreases in osteoclast number (24%, Figure 2(f)) and erosion surface/bone surface (34%, *P* < 0.02, *data not shown*) were observed in dnNFAT^OB^ mice when compared to control mice.

**Figure 3:**
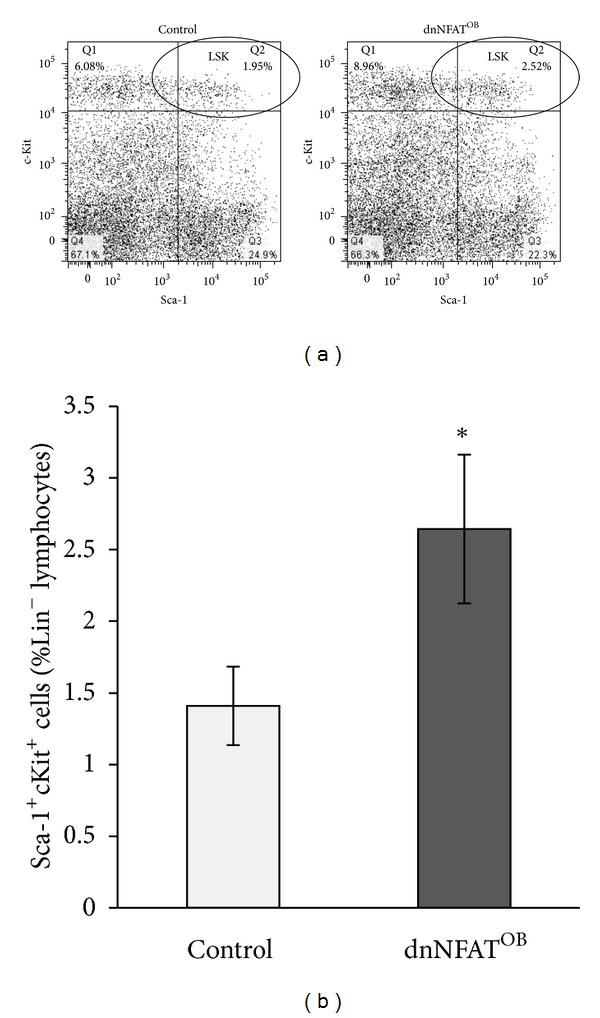
Lin^−^Sca1^+^cKit^+^ (LSK) hematopoietic stem/progenitor cells are increased in dnNFAT^OB^ mice. Bone marrow was flushed from tibiae and femora of 12-week-old control and dnNFAT^OB^ mice and analyzed by flow cytometry for detection of LSK cells. (a) Representative dot plots from control and dnNFAT^OB^ mice showing c-Kit^+^Sca-1^+^ cells gated from the Lin^−^ cell population. (b) Percentages of LSK cells were analyzed from control (*n* = 8) and dnNFAT^OB^ (*n* = 7) mice. Mean indicated by black bar; *P < 0.05 when compared to the control.

**Figure 4:**
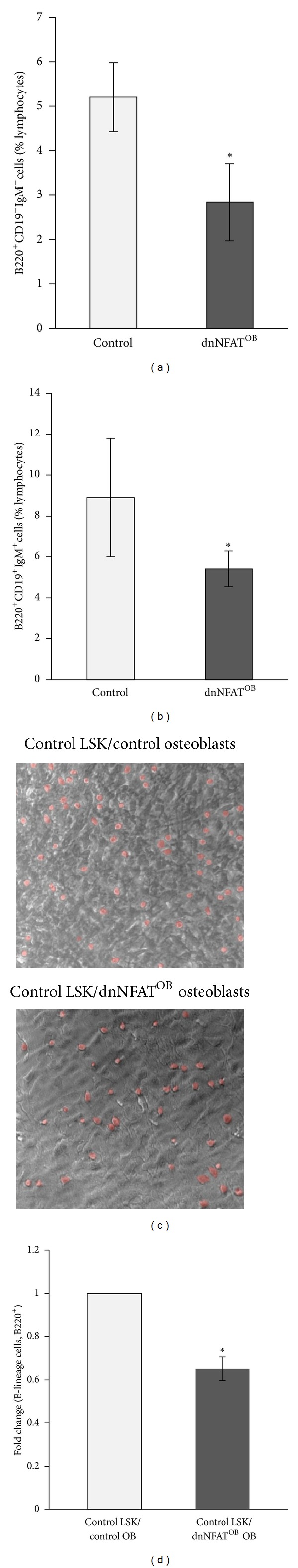
B-cell differentiation is decreased by the inhibition of NFAT signaling in osteoblasts. (a-b) Bone marrow was flushed from tibiae and femora of 12-week-old control and dnNFAT^OB^ mice and analyzed by flow cytometry to examine B-cell development. Percentages of B-lineage cells were determined for control (*n* = 5) and dnNFAT^OB^ (*n* = 3) mice from the gated lymphocyte population. (a) B220^+^CD19^−^IgM^−^ (b) B220^+^CD19^+^IgM^+^. Mean indicated by black bar. (c-d) Primary osteoblasts were harvested from calvariae of 1-day-old control and dnNFAT^OB^ mice and cultured for 14 days in the presence of *β*-glycerophosphate and ascorbic acid-2-phosphate to induce osteoblast differentiation. Bone marrow was flushed from 12-week-old control mice, and LSK cells were sorted by flow cytometry, seeded on differentiated primary osteoblasts, and cocultured for 14 days. At the end of the culture, osteoblasts and hematopoietic cells were trypsinized and analyzed by flow cytometry for the B220 cell marker. (c) Representative phase-contrast images show hematopoietic cells (*indicated by red pseudocolor*) cocultured on primary osteoblasts. (d) B220^+^ B-lineage cells were analyzed by flow cytometry after coculture of LSK cells on control and dnNFAT^OB^ differentiated primary osteoblasts. Values, obtained from three independent experiments performed in duplicate, represent the mean ± SD; *P < 0.05 compared to control.

To assess how NFAT signaling in osteoblasts impacted B-cell development *in vivo*, bone marrow was flushed from tibiae and femora of 12-week-old mice and analyzed by FACS. Analysis of Lin^−^Sca-1^+^cKit^+^ (LSK) cells showed that LSK frequency was significantly increased ~1.8-fold in dnNFAT^OB^ mice when compared to control animals (Figures 3(a) and 3(b)). Examination of B-lineage subsets based on expression of B220, CD19, and surface IgM showed that frequencies of B220^+^CD19^−^IgM^−^ pre-pro B cells were significantly reduced in dnNFAT^OB^ mice (~40% reduction, Figure 4(a)), as were frequencies of B220^+^CD19^+^IgM^+^ immature B cells (40% reduction, Figure 4(c)). These results showed that inhibition of NFAT activation in osteoblasts resulted in a modest increase in LSK cells and inhibited the generation of B-lineage progenitor cells. 

To further establish whether NFAT signaling in osteoblasts plays a role in B-cell differentiation from HSPCs, LSK cells were cocultured with differentiated primary osteoblasts* in vitro*. Calvarial osteoblasts were harvested from 1-day-old control or dnNFAT^OB^ mice and differentiated for 14 days. LSK cells were sorted by flow cytometry from 12-week-old control mice and cocultured on control and dnNFAT^OB^ differentiated primary osteoblasts. After 14 days of coculture, fewer adherent HSPCs were observed in the cocultures on dnNFAT^OB^ osteoblasts compared to HSPCs cocultured on control osteoblasts (Figure 4(c), *indicated by red pseudocolor*). FACS analysis of cells within the wells showed that the frequency of B220^+^ B-lineage cells recovered from LSK/dnNFAT^OB^ osteoblast cocultures was reduced by 35% compared with B220^+^-cell frequencies from LSK/control osteoblast cocultures (Figure 4(d)). The reduced frequency of B220^+^ cells in LSK/dnNFAT^OB^ osteoblast cocultures supports findings *in vivo* that NFAT signaling in osteoblasts plays a role in B lymphopoiesis. The *in vitro* coculture results using purified osteoblasts also suggest that dnNFAT^OB^-mediated inhibition of B-cell development is a cell-autonomous effect.

**Figure 5:**
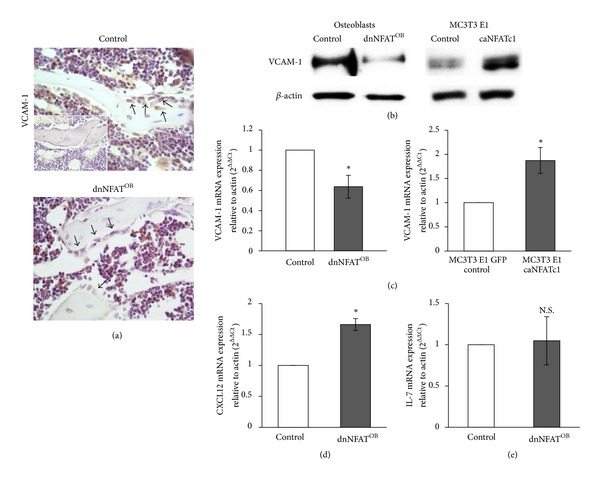
NFAT activation in osteoblasts positively controls VCAM-1 expression. (a) Tibiae were harvested from 12-week-old mice and examined by immunohistochemistry with anti-VCAM-1 (*brown*) and counterstained with hematoxylin (*blue*). Negative control staining was performed by using normal rabbit IgG instead of primary antibody (*top panel inset*). Magnification, 400x. (b-c) Primary osteoblasts were harvested from calvariae of 1-day-old control and dnNFAT^OB^ mice and differentiated for 14 days in the presence of *β*-glycerophosphate and ascorbic acid-2-phosphate to induce osteoblast differentiation. GFP control and ca-NFATc1-expressing MC3T3 E1 cells were cultured for 4 days. (b) Proteins were extracted from control and dnNFAT^OB^ differentiated primary osteoblasts, GFP control, and ca-NFATc1 MC3T3 E1 cells and separated by SDS-PAGE. Immunoblots were developed using antibodies against VCAM-1 and *β*-actin. Immunoblots are representative of three independent experiments. (c–e) RNA was extracted from control and dnNFAT^OB^ differentiated primary osteoblasts ((c), *left panel*) and GFP control and ca-NFATc1 MC3T3 E1 cells ((c), *right panel*), and real-time PCR was performed for (c) VCAM-1, (d) CXCL12, and (e) IL-7 and *β*-actin expression. Values were obtained from three independent experiments and represent the mean ± SD of VCAM-1, CXCL12, or IL-7 levels relative to *β*-actin; *P < 0.05 compared to control.

During B-cell development, pro-B cells bind VCAM-1 on osteoblasts. Studies have shown that NFAT regulates expression of VCAM-1 in different cell types, such as smooth muscle. To examine whether NFAT activation in osteoblasts regulates the expression of VCAM-1 in osteoblasts, immunohistochemistry was performed on tibiae from 12-week-old mice with an antibody directed against VCAM-1. As shown in Figure 5(a), dnNFAT^OB^ mice have reduced levels of VCAM-1 on osteoblast cell surfaces (*indicated by arrows*) when compared to control mice. To confirm that NFAT regulates VCAM-1, gene expression and protein levels of VCAM-1 were examined by real-time PCR and western blotting analyses in primary osteoblasts harvested from control and dnNFAT^OB^ mice as well as MC3T3 E1 preosteoblasts that were transduced with constitutively-active NFATc1 (caNFATc1). The efficiency of overexpressing caNFAT has previously been examined by our group where we show that NFATc1 nuclear translocation is increased by 600% [2, 10]. VCAM-1 gene expression relative to *β*-actin was significantly decreased (36%) in dnNFAT^OB^ primary osteoblasts compared to control primary osteoblasts, and the overexpression of NFATc1 in osteoblasts significantly increased VCAM-1 gene expression when compared to parent MC3T3 E1 cells (~1.9-fold increase; Figure 5(b)). Furthermore, protein levels of VCAM-1 were lower in dnNFAT^OB^ mice when compared to control mice and were dramatically increased in MC3T3 E1 cells that overexpressed NFATc1 (Figure 5(c)). Additionally, CXCL12 and IL-7 gene expression was evaluated in control and dnNFAT^OB^ primary osteoblasts. We discovered that CXCL12 gene expression significantly increases (~60%, Figure 5(d)) in dnNFAT^OB^ osteoblasts when compared to control, while IL-7 expression remains unchanged (Figure 5(e)).

These data demonstrate that NFAT activation in osteoblasts not only negatively regulates osteoblast differentiation and bone formation, but also decreases B-cell development in the bone marrow microenvironment. In addition, these results indicate that NFAT activation in osteoblasts may play a role in the expression of VCAM-1, which has been shown to be critical for HSPC differentiation into B-lineage cells.

## 4. Discussion

The calcineurin/NFAT signaling pathway in osteoblasts has been previously shown to negatively regulate osteoblast differentiation *in vitro* and bone volume *in vivo* [2, 3]. Pharmaceutical agents targeting this pathway in osteoblasts may provide treatment options for patients suffering from bone diseases, such as osteopenia and osteoporosis [29]. NFAT is an essential transcription factor for the expression of various genes, but it is relatively unknown how NFAT activation in osteoblasts affects the hematopoietic niche in the bone marrow microenvironment. It has been shown that NFATc2^−/−^ mice (C57BL/6 background) exhibit increased bone formation and decreased populations of granulocytes, lymphocytes, and megakaryocytes in the bone marrow [30]; however, this NFAT knockout mouse model was not an osteoblast specific model. To determine whether inhibition of NFAT activation specifically in osteoblasts regulates hematopoiesis in the bone marrow microenvironment, we generated a mouse model expressing dominant-negative NFAT driven by a 2.3 kb fragment of the collagen *α*I promoter to inhibit the transactivation of NFAT 1–4 isoforms in osteoblasts [25]. Analysis of transgenic mice showed that inhibition of NFAT signaling in osteoblasts significantly increased the frequency of HSPCs and decreased the frequencies of B-lineage cells in bone marrow. Furthermore, blocking NFAT activation in osteoblasts decreased the ability of HSPCs to develop into B220^+^ B-lineage cells *in vitro*.

Mice containing the 2.3 kb fragment of the ColI-dnNFAT transgene did not show any significant differences in size, weight, and survival rate when compared to control littermates; however, dnNFAT^OB^ mice displayed significant increases in bone formation parameters. These results are consistent with our previous findings showing increased bone volume after treatment with low concentrations of cyclosporin A, a calcineurin inhibitor that prevents NFAT dephosphorylation [10]. Similarly, this bone phenotype was also observed in mice with a genetic deletion of Cnb1 in osteoblasts [3]. Normally, resorbed bone is replaced by new bone, but dnNFAT^OB^ mice showed increased numbers of osteoblasts and decreased numbers of osteoclasts and erosion surface, suggesting that the increase in bone formation may be due to either an increase in recruitment of osteoblasts at the bone surface or a decrease in bone resorption due to a lower number of osteoclasts. 

NFAT transcription factors are involved in the differentiation and development of various cell types, including osteoblasts. Many studies have shown that the number of HSCs in the bone microenvironment is positively correlated to the number of osteoblasts and vice versa [31, 32]. The role of NFAT in cellular interactions between osteoblasts and hematopoietic lineage cells in the bone microenvironment is relatively unknown. Studies have examined NFAT signaling in the bone microenvironment using animal models that have global NFAT inhibition or by the inhibition of NFAT activity exclusively in hematopoietic cells [30, 33]. It has been shown that adult NFATc2 knockout mice exhibit extramedullary hematopoiesis and suffer from osteomyelosclerosis and hypoplasia in the bone marrow (with significant losses of erythrocytes, lymphocytes, and megakaryocytes) [30]. Others have reported that repression of NFAT signaling in HSCs negatively regulates the development of myeloid progenitor cells [33]. In the present study, we demonstrate that inhibition of NFAT activity specifically in osteoblasts may impact the hematopoietic stem cell niche and can interfere with B lymphopoiesis in the bone marrow microenvironment. Previous studies have shown that osteoblast number is positively correlated to numbers of HSPCs in the bone marrow due to cellular communication between osteoblasts and HSPCs [12, 34], suggesting that the increased number of HSPCs may be consequential to the increased number of osteoblasts observed in the dnNFAT^OB^ mice. 

B lymphocytes develop and mature in the bone marrow in close association with osteoblasts and/or stromal cells. In dnNFAT^OB^ mice, there was a significant decrease in the pre-pro B-cell subset, suggesting that NFAT activity in osteoblasts is critical for specification and/or commitment to the B-cell lineage. No difference was observed in the number of bone marrow cells in dnNFAT^OB^ mice, suggesting that the change in the number of B-lineage cells is not likely due to a change in bone marrow cellularity. NFAT regulation of VCAM-1 expression on osteoblasts may be partially responsible for this phenotype, although Cn/NFAT-regulated chemokines, such as CXCL12, and other membrane receptors are also important for promoting B-cell development. During early stages of B lymphopoiesis, hematopoietic progenitor cells are known to bind to stromal cells via VLA-4/VCAM-1 interactions at the cell surface [14, 16, 20]. NFAT transcription factors have been previously shown to regulate the expression of VCAM-1 in other cell types [23, 24], and blocking VCAM-1 in osteoblasts has resulted in decreased B-cell development [20, 21]. Previous studies examining the role of NFAT signaling in CXCL12 expression where treatment with cyclosporin A, an inhibitor of Cn/NFAT signaling, increased the gene expression and protein levels of CXCL12 in decidual stromal cells [35]. Additionally, treatment with CXCL12 interrupted the Cn/NFAT pathway in rat cardiac myocytes [36], suggesting a negative feedback loop. Others have suggested that IL-7 mediates the activity of NFAT [37, 38], but little information is known about the role NFAT signaling plays in IL-7 expression. Our results demonstrate that gene expression and protein levels of VCAM-1 in dnNFAT^OB^ osteoblasts are significantly reduced when compared to normal osteoblasts, suggesting that NFAT regulates VCAM-1 expression in osteoblasts at the transcriptional level and that reduced expression of this receptor may negatively affect B-cell development. Increased gene expression of CXCL12 in dnNFAT^OB^ osteoblasts is consistent with previous work but may not influence the observed B-cell phenotype.

## 5. Conclusions

We report that the inhibition of NFAT activation in osteoblasts increases osteoblast differentiation and bone formation and decreases B-cell development in the bone marrow microenvironment. These data suggest that long-term NFAT inhibition in osteoblasts may result in a weakened immune status due to decreased development of B-lineage cells in the bone marrow. The decreased expression of VCAM-1 in osteoblasts that can result from blocked NFAT signaling could be a contributor to this phenotype.
